# Uterine Transplantation Using Living Donation: A Cross-sectional Study Assessing Perceptions, Acceptability, and Suitability

**DOI:** 10.1097/TXD.0000000000001124

**Published:** 2021-02-18

**Authors:** Benjamin P. Jones, Abirami Rajamanoharan, Nicola J. Williams, Saaliha Vali, Srdjan Saso, Ifigenia Mantrali, Maria Jalmbrant, Meen-Yau Thum, Cesar Diaz-Garcia, Sadaf Ghaem-Maghami, Stephen Wilkinson, Isabel Quiroga, Peter Friend, Joseph Yazbek, J. Richard Smith

**Affiliations:** 1 West London Gynaecological Cancer Centre, Hammersmith Hospital, Imperial College NHS Trust, London, United Kingdom.; 2 Department of Surgery and Cancer, Imperial College London, London, United Kingdom.; 3 Department of Politics, Philosophy and Religion, Lancaster University, Lancaster, United Kingdom.; 4 Lister Fertility Clinic, The Lister Hospital, London, United Kingdom.; 5 The Oxford Transplant Centre, The Churchill Hospital, Oxford University Hospitals NHS Trust, Oxford, United Kingdom.

## Abstract

A uterine transplantation is a nonvital, quality-of-life–enhancing solid organ transplant. Given improvements in donor risk profile and the anticipated shortage of suitable deceased donors, nondirected donation could facilitate sustainability as uterine transplantation moves from research into the clinical realm. The aim of this article is to determine perceptions and identify motivations of potential nondirected living uterus donors and assess acceptability and suitability.

**Methods.:**

A cross-sectional survey using an electronic questionnaire among women who have inquired about donating their uterus for uterine transplantation.

**Results.:**

The majority of respondents “strongly agreed” or “agreed” that the most prevalent motivations to donate their uterus include helping someone carry and give birth to their own baby (n = 150; 99%), helping others (n = 147; 97%), and because they no longer need their womb (n = 147; 97%). After considering risks of uterus donation, the majority were still keen to donate their uterus (n = 144; 95%), but following a process of exclusion using donor selection criteria, less than a third (n = 42; 29%) were found to be suitable to proceed.

**Conclusions.:**

This study demonstrates novel insight into the motivations of women who wish to donate their uterus and displays high levels of acceptability after consideration of the risks involved. Despite the physical risk and transient impact upon ability to undertake activities of daily living, women who donate their uterus expect to gain psychological and emotional benefits from enabling another woman to gestate and give birth to their own future children. However, currently used selection criteria reduce the number of potential donors significantly.

## Abstract 2

Supplemental Digital Content is available in the text.

## INTRODUCTION

The first live birth following uterine transplantation (UTx) was reported in 2014.^1^ More than 70 cases have now been performed worldwide, and outcomes have recently been reported from 45 cases, which have yielded 9 live births so far.^2^ UTx has thereby been proven a feasible fertility restoring intervention for women with absolute uterine factor infertility, allowing them to conceive and gestate their future children. UTx is a nonvital, quality-of-life–enhancing solid organ transplant. The nonessential nature of a uterus after the completion of one’s family allows the opportunity to use both living donors (LDs) and deceased donors (DDs). Each donor type presents ethical, logistical, physiological, immunological, and anatomical advantages and disadvantages that continue to stimulate debate.^3,4^ LD can either be directed or nondirected in nature. Directed organ donations include the donation of an organ that is intended for a specific individual, from someone with an emotional or biological relationship, such as a friend or close family member. Conversely, nondirected donations are from donors with no such preexisting relationships and are unknown to the recipient.

The greatest advantage of DD is that whereas LD necessitates and risks significant physical and psychological harms to donors, DD completely avoids these harms and risks. In LD UTx cases so far, >1 in 10 donors have suffered a complication necessitating further surgical intervention,^2^ including ureteric injuries,^5-7^ fecal impaction requiring digital evacuation under anesthesia,^8^ and vaginal cuff dehiscence.^9^ Around a quarter of donors have suffered less serious complications including wound infection,^7^ bladder hypotonia,^6^ urinary tract infection,^2^ leg/buttock pain, depression,^9^ intubation-related respiratory failure, and anemia.^2^ The risks associated with LD are however expected to diminish over time as surgical techniques and selection criteria are finessed. Recent modifications to the vascular dissection process have, for example, reduced surgical risks and operative times,^2^ by prioritizing ovarian venous drainage when possible,^6,9-11^ as opposed to the uterine venous complex.^7^ Similarly, the implementation of minimally invasive surgical techniques in recent cases offers further risk reduction and an enhanced recovery process.^11-13^

The ethics literature on UTx discusses the risks associated with LD as well as concerns regarding consent and the potential for donor regret.^14-16^ This has led many to conclude that DD is preferable provided that there are sufficient donor uteri, similar success rates, and that retrieval does not threaten the viability of lifesaving organs for transplant.^17-19^ When these conditions are *not* met, however, the use of LDs may be justified provided that the donor provides informed and voluntary consent, the risks are proportionate to potential benefits *and* fall below an acceptable absolute threshold, and risks to the donor are minimized.^17^ This is similar to the approach adopted in other solid organ transplants that use LD such as kidney and liver lobe transplantation.

Given improvements in donor risk profile and the anticipated shortage of suitable DDs,^20^ a combination of both LD and DD will likely be needed in short to medium term.^21^ In the context of LD, reliance solely on directed donations could exclude potential recipients who do not have a willing and suitable donor among their friends or family members. Therefore, as is the case in other solid organ transplants, allowing nondirected altruistic donation from donors previously unknown to the recipient could facilitate sustainability as UTx progresses from research into the clinical realm. However, nondirected living organ donation raises ethical concerns regarding the potential for undue inducement and defective understanding.^22^ Despite this, some scholars have suggested that nondirected donation could be preferable to directed donation because the use of donors known to recipients brings with it the risk of undue pressure to donate from family members or friends.^16,23^

In light of this, the primary aim of this study was to identify the perceptions of potential donors toward uterus donation and evaluate their motivations underpinning their desire to donate. Second, investigation of their suitability, as well as determination of acceptability and willingness to proceed were also evaluated, after disclosure and discussion of the risks associated with donation, expected recovery period and process, and its potential impact upon their lives.

## MATERIALS AND METHODS

### Study Design

This study was developed as a direct response to demand from women who contacted Womb Transplant UK to inquire about the possibility of uterus donation. All women who contacted Womb Transplant UK (registered charity no. 1138559) to enquire about donating their uterus between November 1, 2014, and November 1, 2019 were invited to participate. An email containing a link to an electronic questionnaire, as well as 2 information leaflets, was sent to potential participants. One information leaflet detailed the conduct of the study, whereas the other explained the proposed process of LD UTx (Appendix S1, SDC, http://links.lww.com/TXD/A312). The questionnaire contained an initial 4-question consent process, which required agreement before proceeding to the 58-question questionnaire (Appendix S2, SDC, http://links.lww.com/TXD/A312). The online platform SurveyMonkey was used to distribute the questionnaire over a 6-mo period between June 1, 2019 and November 31, 2019. Initial questions elicited demographic information before determining background medical, surgical, psychiatric, obstetric, gynecological, and social history. Further questions ascertained perceptions of adoption, surrogacy, and knowledge and opinions of UTx. Subsequent questions specifically assessed acceptability of the necessary preoperative investigations, the potential need for hormone supplementation in postmenopausal donors, and willingness to donate *after* considering the risks and expected recovery time of uterus donation. The final questions determined knowledge and perceptions of potential recipient risk, views on the provision of updates regarding recipient progress posttransplant, and financial considerations. Most questions were closed, using tick boxes, with the option to include further comment in cases when further description was warranted. Likert scales were used in questions related to perceptions. A number of women were consulted in the creation of the information leaflets and questionnaire to help refine questions, content, and phraseology. The questionnaire was further piloted among a sample of potential donors to assess understanding and readability.

### Ethics Approval

Ethics approval was received from Imperial College Research Ethics Committee on May 30, 2019 (reference no: 19IC5202).

### Data Analysis

SPSS version 24 software (SPSS, Chicago, IL) was used for analysis. Descriptive statistical analysis was described as mean ± SD or median ± range. The Likert scale responses were quantified using a weighted ranking system to ascertain the most influential perceived factor (0 = not at all; 4 = definitely).

## RESULTS

Two hundred eighty-five women approached the charity regarding the possibility of donating their uterus. Of them, 152 women subsequently completed the questionnaire, resulting in a response rate of 53.3%. The cohort comprised 151 women and 1 female to male transgender man. The demographics of the respondents are summarized in Table 1. The most prevalent age group was 30–39 y (n = 50; 33%). The majority of respondents were Caucasian (n = 143; 94%), fluent in English (n = 151; 99%), married (n = 76; 50%), and in either full-time/part-time employment or self-employed (n = 103; 68%). A minority (n = 24; 16%) were current smokers, 30% were ex-smokers (n=45), and the remainder had never smoked (n = 83; 55%).

**TABLE 1.** Basic demographic information of uterine donor cohort

			Number (n)	%
Age (y)	16–19		2	1
	20–29		36	24
	30–39		50	33
	40–49		43	28
	50–59		14	9
	60+		7	5
Body mass index (kg/m^2^)	<18.4	Underweight	5	3
	18.5–24.4	Normal	54	36
	25–29.9	Overweight	45	30
	30–34.9	Obesity I	27	18
	35–39.9	Obesity II	11	7
	>40	Obesity III	10	7
Ethnicity	White		143	94
	Asian		4	3
	Black		2	1
	Mixed		2	1
	Other		1	1
Language	English		143	94
	Non-English but fluent in English		8	5
	Non-English but not fluent in English		1	1
Religion	Christianity		43	28
	Hinduism		2	1
	Islam		2	1
	Atheism		61	40
	Other		31	20
	Would rather not say		13	9
Employment status	Employed full time		57	38
	Employed part time		26	17
	Self-employed		20	13
	Housewife		26	17
	Unemployed		12	8
	Student		5	3
	Would rather not say		6	4
Relationship status	Single		43	28
	Living with partner		19	13
	Married		76	50
	Divorced		9	6
	Separated		2	1
	Would rather not say		3	2
Smoking status	Never smoked		83	55
	Ex-smoker		45	30
	Current smoker		24	16
Alcohol intake (U/wk)	0		69	45
	≤14		79	52
	>14		4	3

### Medical History

Table 2 summarizes the obstetric background of our cohort. Over a third were nulliparous (n = 56; 37%). Of those who had children previously (n = 96), around three-quarters (n = 70; 73%) delivered their children vaginally, whereas the remainder had at least 1 child born by cesarean section (n = 26; 27%). Of those who had previously had cesarean sections, 13% (n = 12) had 1, 11% (n = 11) had 2, and 3% had ≥3. More than three-quarters of those with previous pregnancies had not experienced significant antenatal or postnatal complications (n = 74; 77%). The most frequently encountered obstetric complication was preterm delivery, which impacted 11% (n = 11).

**TABLE 2.** Obstetric history of study cohort

		Number (n)	%
No. of miscarriages (n = 152)	0	115	76
	1	19	13
	2	10	7
	3+	8	5
No. of children (n = 152)	0	56	37
	1	11	7
	2	36	24
	3	30	20
	4+	19	13
No. of cesarean section (n = 96)	0	70	73
	1–2	23	24
	>2	3	3
Problems encountered in pregnancy (n = 96)	No problemsPreterm delivery <37 wkPreeclampsiaGestational diabetesHeavy bleeding after deliveryObstetric cholestasis	74113422	77113422

Around a quarter of our cohort had previously experienced miscarriages (n = 37; 24%), and 5% (n = 8) had experienced ≥3. Seventeen percent (n = 26) of respondents were postmenopausal. Of those still menstruating, 84% (n = 106) reported regular cycles and 16% (n = 20) reported irregular cycles. Nearly a quarter of participants had previous abnormal cervical cytology (n = 34; 22%). Fifty percent of this number subsequently underwent surgical treatment (n = 17), and 50% were monitored conservatively (n = 17) and 97% (n = 33) had normal cytology since.

The mean body mass index of participants was 28.4 kg/m^2^ ± 8.56. Sixty-one percent (n = 93) did not report any significant medical history. The previous medical, psychological, and abdominopelvic surgical history of participants is summarized in Table 3. Only 1 individual reported a previous history of malignancy, which affected her breast. None of the women in this cohort were known to be HIV positive or to have syphilis or hepatitis. Around half reported other minor medical conditions, which would not impact suitability to donate their uterus (n = 29; 49%). Over three-quarters of participants did not have any previous psychiatric history (n = 116; 76%). The majority of the cohort had not previously undergone abdominopelvic surgery (n = 90; 59%).

**TABLE 3.** Medical, psychological, and surgical history of study cohort

		Number (n)	% of cohort
Previous medical history	No medical issues	91	61
	Asthma	12	8
	Hypertension	4	3
	Diabetes	4	3
	Thyroid disorders	11	7
	Clotting disorder	1	1
	Neurological disorders	3	2
	Rheumatology disorders	10	6
	Inflammatory bowel disease	1	1
	Hepatobiliary disorders (minor)	2	1
	Previous malignancy	1	1
	Other minor medical disorders	29	19
Previous mental health history	No mental health issues	116	76
	Depression	26	17
	Bipolar	2	1
	Generalized anxiety	18	12
	Posttraumatic stress disorder Obsessive	5	3
	compulsive disorder borderline	1	1
	Personality disorder	5	3
	Autism	2	1
Previous abdominal or pelvic surgery	No previous abdominopelvic surgery	90	59
	1× cesarean section	10	6
	2× cesarean section	11	7
	3× cesarean section	3	2
	1× exploratory laparotomy	1	1
	1× laparoscopy	28	18
	2× laparoscopy	2	1

### Understanding and Perceptions of Uterine Transplantation

Although 79% (n = 120) of individuals “strongly agreed” or “agreed” that adoption and surrogacy were suitable methods to have children, all participants (n = 152; 100%) felt that women should be freely able to voluntarily donate their uterus for UTx if adequately informed of the process. The majority of respondents reported knowing “a fair amount” or “a lot” about UTx (n = 8; 58%), around a third had “heard it discussed only a few times” (n = 52; 34%), and a minority admitted to “knowing nothing” (n = 12; 8%). The majority of individuals, however, “strongly agreed” or “agreed” that they understood the potential benefits (n = 143; 94%) and risks (n = 131; 86%) of uterus donation.

Nearly all respondents “strongly agreed” or “agreed” that they would consent to undergoing the necessary preoperative investigations (n = 150; 98%) and held the expected recovery process, including a 4-d inpatient admission and an 8-wk recovery period, to be acceptable (n = 148; 97%). All but 1 respondent understood that future pregnancies in the recipient would not be genetically related to the uterus donor (n = 151; 99%). All participants reported completion of their family (n = 152), with over a third of these being nulliparous (n = 55; 36%). All premenopausal respondents understood that they would be unable to gestate a pregnancy after uterus donation (n = 126). The majority of these understood that the intended operation involved ovarian conservation, which would result in normal hormone levels postoperatively (n = 121; 96%). Ninety-six percent of respondents (n = 122) “strongly agreed” or “agreed” that donation of their uterus would cease future menstruation and the majority “strongly agreed” or “agreed” that cessation of menstruation would improve their quality of life (n = 113; 89%). Of those participants who were postmenopausal (n = 26), the majority “strongly agreed” or “agreed” that they would accept 3–6 mo of hormone replacement therapy to demonstrate endometrial function by inducing withdrawal bleeds preoperatively (n = 24; 92%).

### Motivations for Uterine Donation

**FIGURE 1.**
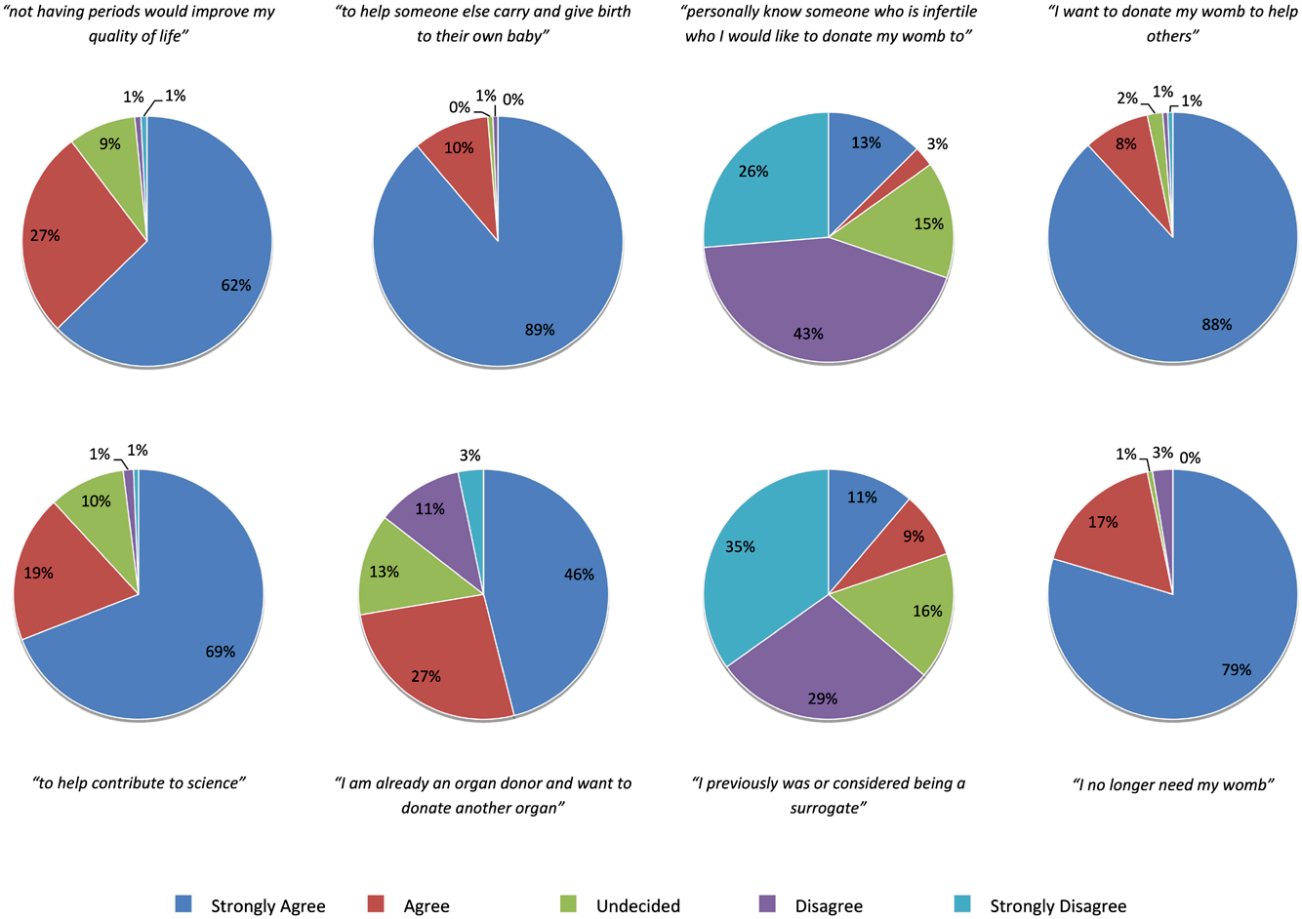
Motivating factors for consideration of uterine donation.

**FIGURE 2.**
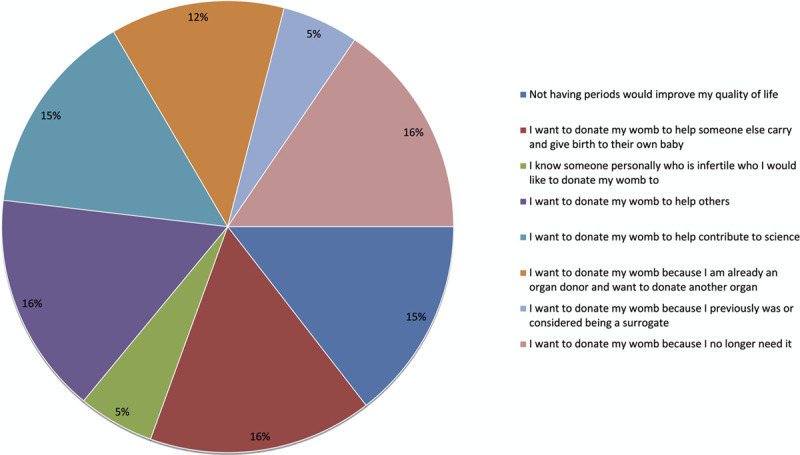
Perceived importance of each factor influencing motivation to donate.

Factors motivating consideration of uterus donation are depicted in Figure 1. The overwhelming majority of respondents “strongly agreed” or “agreed” that they were motivated to donate to help someone else carry and give birth to their own baby (n = 150; 99%), help others (n = 147; 97%), and because they no longer need their womb (n = 147; 97%). Nearly three-quarters of the cohort “strongly agreed” or “agreed” that they were already organ donors and wanted to donate another organ (n = 110; 72%). Twenty percent (n = 30) “strongly agreed” or “agreed” that they wanted to donate because of previously considering or being a surrogate. Only 15% (n = 23) of the cohort “strongly agreed” or “agreed” to personally knowing someone infertile who they wanted to donate their uterus to. Using a weighted scoring system, Figure 2 represents the importance respondents placed on each influencing factor. The 2 most influential factors were the desire to help someone else carry and give birth to a child and wanting to help others. The least influential factors were personally knowing someone who was infertile that they wanted to donate to and wanting to donate because of considering or being a surrogate previously.

More than three-quarters (n = 117; 77%) of respondents were aware that UTx recipients would be at greater risk of malignancy and infection because of the immunosuppressive medications required to be taken postoperatively. However, just 9% (n = 4) agreed or strongly agreed that this knowledge would impact their decision to donate. More than two-thirds of the cohort wanted to be kept informed of the future progress of recipients of their uteri (n = 105; 69%), around a quarter (n = 40; 27%) were undecided, and 3% (n = 5) would not want to know. Most respondents “strongly agreed” or “agreed” to understanding they would not receive any payment for donating their uterus (n = 145; 95%); however, 4 (2%) individuals “strongly disagreed” or “disagreed.” Given the likely media interest in the process due to its novelty, the majority (n = 129; 85%) “strongly agreed” or “agreed” that uterus donation would risk unwanted media attention.

After reading the information and considering the risks, nearly all participants were still keen to donate their uterus (n = 144; 95%). Eight (4%) remained undecided, with the majority expressing a desire to know further information. One participant, however, disagreed, citing the desire for further counseling to help not develop an attachment to the recipient’s family.

### Suitability

**FIGURE 3.**
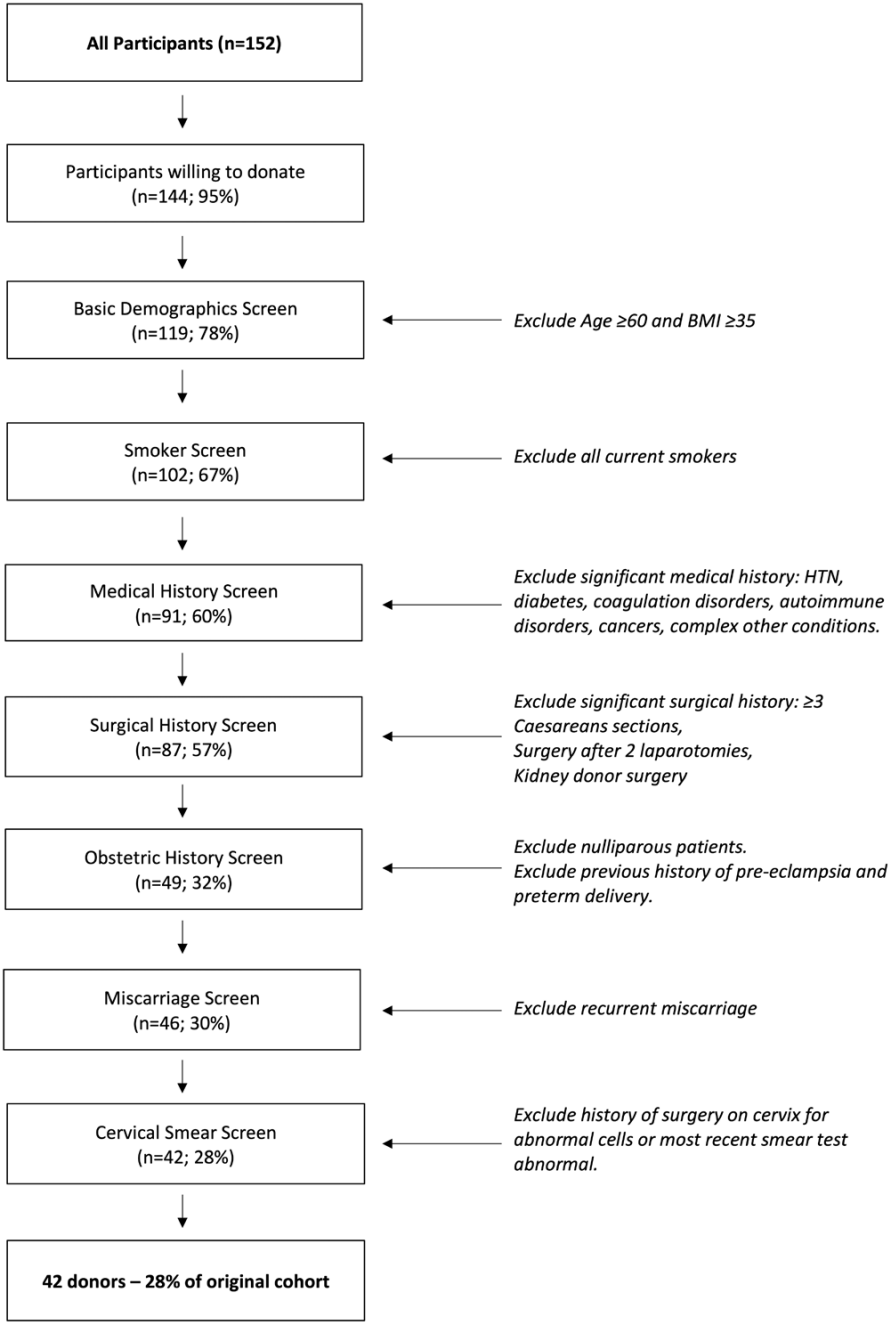
Systematic process of exclusion using the UK uterine transplant living donor selection criteria. BMI, body mass index.

Figure 3 summarizes a systematic process of exclusion, using the donor selection criteria we have implemented for the UK UTx LD program (Table 4). In accordance with this criterion, 25 (17%) respondents would be excluded from uterus donation because of age ≥60 y or body mass index >35 kg/m^2^. Seventeen (12%) more would be excluded for being current smokers. A further 11 (8%) would be excluded because of significant preexisting medical conditions and 4 (3%) more would be excluded because of multiple or significant previous surgical history. More than a quarter (n = 38; 26%) would not be eligible because of nulliparity, previous preeclampsia, or preterm delivery. Three (2%) respondents would be considered unsuitable because of recurrent miscarriage, and 4 (3%) would be excluded because of previous cervical surgery for abnormal cells or a recent abnormal cytology result. Following the implementation of the selection criteria, 42 respondents (29%) would be considered suitable to proceed with further clinical evaluation, counseling, and further investigation to determine suitability to donate.

**TABLE 4.** Donor screening criteria for UK living donor uterine transplantation program

Inclusion	Aged 18–60 y
	Multiparous
	BMI <35 kg/m^2^
Exclusion	History of cancer
	Previous multiple/significant uterine surgery
	2× cesarean section acceptable
	No previous myomectomies
	Previous significant cervical surgery (cone biopsy or LLETZ)
	HPV positive or abnormal cervical cytology
	Significant systemic disease (diabetes, hypertension, autoimmune conditions, etc)
	Previous obstetric problems including preeclampsia and delivery <37/40
	Previous recurrent miscarriages (≥3)
	Current IV drug abuse
	Active bacteremia/fungemia
	Active smoker

BMI, body mass index; IV, intravenous; LLETZ, large loop excision of the transformation zone.

## DISCUSSION

The data presented herein demonstrate novel insight into the perceptions of potential UTx LDs and portray robust and diverse motivations and desires to proceed with the donation process following an understanding of the risks involved and a realistic awareness of the expected process and recovery. This reinforces the generally supportive previously reported public perceptions of UTx. When considering the public, a web-based questionnaire completed by 1247 US residents identified that more than three-quarters (78%) supported UTx.^24^ Similar levels of support were seen in a European study including 2000 Swedish women, wherein 80% were found to be in favor of UTx and considered it to be more acceptable than surrogacy.^25^ However, a study in 3098 women of reproductive age in Japan demonstrated less favorable support, with just 44.2% being in favor of UTx. However, 47.5% had no opinion, which highlights a potential lack of awareness of UTx in those surveyed, particularly when the vast majority (93.3%) felt that UTx should be permitted either with or without further discussion.^26^ In the context of DD, more than a decade ago, before the first live birth following UTx was reported,^1^ just 6% of donor families agreed to uterine procurement as part of deceased donation.^27^ However, 10 y later, attitudes to uterine donation appear to have improved, with three-quarters of women agreeing to donation of their uterus at the time of their death, which increased to 87% after becoming informed about the purpose of the transplant.^28^ In addition, a recent European study demonstrated that donation of the uterus was readily accepted, with no refusals observed in 7 cases.^29^

In the context of LD, it is essential to understand prospective donors’ motivations and expectations to ensure comprehension of the harms, risks, and likely benefits of the procedure on the part of the donor and that willingness to donate is not coerced or induced, for example, with expectation of payment. As shown in the data presented herein, nearly all respondents appeared to understand that they would *not* receive payment for their donation, which is reassuring. Prospective LDs should be nonetheless provided with clear information regarding the financial implications of a decision to donate such as the prohibited status of payment for organs *and* any reimbursement they may claim to offset the costs of donation.

If of childbearing age, potential donors’ future reproductive plans need to be explored, as does the possibility that their aspirations and plans could change if they met a new partner in future. We demonstrate that, of those included in the survey, the most common motivation for donation was to help someone else carry and give birth to a child, closely followed by a desire to help others, which is consistent with the primary reported motivations underpinning donation in the first 6 nondirected UTx donors in the Dallas UtErine Transplant Study (DUETS).^30^ Moreover, this is in line with motivations expressed in the nondirected donation of other organs, such as kidney donation, wherein the desire to donate has been described by donors as “compelling,” and donation is associated with significant psychological motives and gains.^31^ Qualitative interviews with nondirected kidney donors identified that donors want to offer someone else a chance at a normal life^32^ and that the altruistic value gained from the gift of donation outweighs the fears and risks associated with the surgery.^33^

Our findings, however, also suggest that the underpinning motives of potential living uterus donors may be mixed, with further consideration of self-regarding interests. A significant majority of respondents (89%) “agreed” or “strongly agreed” that the cessation of menstruation would improve their quality of life, suggesting that they would expect physical benefits from donation. This inference is supported by work exploring the motivations of living organ donors more generally, which shows that, despite the narratives often imposed on them, “many organ donors are not pure altruists: willing to sacrifice their own interests for the sake of another with no expectation of, or desire for, benefit … they hold mixed and complicated motivations for donation.”^22^

We also clearly demonstrate the anticipated decline in numbers of potential donors in the context of using proposed standard donor criteria. These figures can be used to estimate eventual donor availability and also manage expectations in potential UTx donors. In DUETS, 79 women initially expressed interest in donating their womb and 62 were subsequently screened by telephone.^34^ Following prescreen, 30 donors completed a detailed health history questionnaire, in a similar fashion to our study, in which 12 women eventually proceeded to be clinically evaluated.^34^ This presents an overall conversion from screening to being clinically evaluated of 19%, which compares with 28% in our cohort. In DUETS, following clinical evaluation, 50% of potential donors eventually proceeded with donation (n = 6) due to a combination of unsuitability or self-withdrawal. Thereby, within our cohort, using a 50% dropout rate, it would be anticipated that approximately 21 women may eventually be suitable to proceed with donation from the initial 152 women.

In UTx cases performed internationally so far, the mean age is 44 y, and the mean age of the potential donors evaluated in DUETS was 40 y.^2^ Although the precise ages were not elicited in this dataset, as 61% of the cohort were aged between 30 and 50 y, they appear broadly similar. In UTx cases performed so far, the vast majority (93%) have had children previously, which is likely to have been skewed following multiparity being an initially recommended inclusion criteria for donors, as it is the only way to demonstrate uterine functionality with certainty.^35^ However, 90% of the potential donors evaluated in DUETS were also multiparous, which is significantly higher than the 63% demonstrated herein. This identifies that despite more than a third of our cohort not having children themselves, they still had insight into the potential benefits of donating their uterus to someone who desires children.

Although the use of marginal organs is acceptable in lifesaving organ transplants, owing to the significant risk of death while on transplantation waiting lists,^36^ it is harder to justify the use of suboptimal grafts in quality-of-life–improving transplants, such as UTx. However, it is currently difficult to determine which donor selection criteria result in a suboptimal graft. Although various selection criteria have been used in trials to date, ongoing discussion in the context of outcomes following UTx is required to determine which criteria optimize graft quality and which criteria are of negligible benefit and therefore unnecessarily limit donor availability. Recent donor criteria have been suggested for implementation in the context of DD.^37^ If extrapolated into LD, their suggested standard donor criteria, which excludes women with miscarriage or cesarean section, would reduce the number of suitable donors to just 25, before comprehensive clinical evaluation and investigation, representing just 16% of the initial cohort. Given that the majority of miscarriages are sporadic and because of problems completely unrelated to the uterus, such as embryonic aneuploidy,^38^ and that 15%–20% of women experience miscarriage,^39^ accepting donors with previous sporadic miscarriages could increase donor availability without detracting from the quality of the graft. Moreover, given that around a quarter of deliveries in the United Kingdom are performed via cesarean section, removing this as an exclusion would improve donor availability.

This is the largest study of its kind to determine the background, perceptions, and motivations of potential UTx LDs, and the first of its kind in a UK population. Its findings demonstrate that nondirected altruistic donation could be a solution to the anticipated shortage of DDs, and lack of availability of living-related donors for women with absolute uterine factor infertility. A weakness of this study includes the fact that participants were self-selected, because they all directly contacted Womb Transplant UK to inquire about uterine donation. As such, the results presented herein are not directly applicable to all women but relate specifically to women interested in donating their uterus. Additionally, the exclusive use of self-reported data and closed-ended questions introduces the potential for bias.

Altruistic nondirected LD UTx donation appears to be ethically and medically acceptable, provided that comprehensive physiological and psychological donor evaluation is undertaken preoperatively, donor risk is minimized as much as possible, and donors are appropriately counseled, thereby facilitating informed consent. As suggested by the findings presented here, donating an organ can generate a number of psychological and emotional benefits and most potential UTx donors express a desire to give another woman the opportunity to bear a child herself. Moreover, in the context of the reported outcomes in cases performed to date, albeit limited in number, it is reassuring that psychological outcomes appear excellent and that little regret has so far been reported.^8,40^

Although the use of DD negates donor risk, the associated logistical difficulties are so complex that they can compromise the viability of UTx programs.^4^ Moreover, even in healthcare systems with well-established DD programs, concerns have been expressed about the potential availability of donors to meet future demand.^21,37^ Although increasing donation after brain death conversion rates is essential, increasing potential DD supply may also be possible by considering uterine donation after circulatory death. However, this is a prospect that requires further research to determine whether the prolonged warm ischemic time in such cases is detrimental to UTx outcomes. As demonstrated herein, given the willingness of some women wishing to donate their uterus to an unknown recipient, the use of nondirected UTx donors is a readily available donor pool that should be explored further.

## CONCLUSION

This study provides novel insight into the motivation of women who wish to donate their uterus to a previously unknown recipient and displays high levels of acceptability after consideration of the risks and expected recovery. Despite the physical risk and expected lengthy recovery process, women who donate their uterus expect to gain psychological and emotional benefit by enabling another woman to bear a child. In the context of expected shortages in DD availability, unnecessarily ruling out nondirected LD is not ethical nor is it sustainable. As such, consideration of the use of nondirected altruistic living donation offers an alternative, following comprehensive medical and psychological assessment and extensive counseling. However, as demonstrated here, despite the desire and motivation to donate, the selection criteria currently being implemented reduce the number of potential donors significantly. Although enhancing outcomes remains paramount, further work is needed to validate selection criteria to optimize donor availability without impacting graft quality.

## ACKNOWLEDGMENTS

This research was supported by a Wellcome Trust Senior Investigator Award (grant097897/Z/11/Z) and a Leverhulme Early Career Fellowship (grant ECF-2018-113).

## Supplementary Material


