# Supplementary material for: Uterine Transplantation Using Living Donation: A Cross-sectional Study Assessing Perceptions, Acceptability, and Suitability
Source: Transplant Direct. 2021 Feb 18;7(3):e673. doi: 10.1097/TXD.0000000000001124 (PMC8183710; doi:10.1097/TXD.0000000000001124)
Supplement: Supplementary file 1 [file txd-7-e673-s001.pdf]

Appendix S1. Womb Transplant donor information (V1 30-4-19)

Background information

Uterine transplantation is a potential treatment for women who cannot become pregnant or maintain pregnancy due to either not having a womb, or having a womb that is damaged or not functional. This affects one in 500 women of childbearing age. The current options to acquire motherhood for these women include adoption or surrogacy. However, not only are these options associated with complex legal, financial, cultural, ethical and religious factors, but they do not offer the women to experience gestation. Uterine transplantation provides an opportunity to overcome these issues whilst also giving these women the opportunity to conceive and carry pregnancy themselves.

More than 60 procedures have now been performed worldwide, and at least 15 babies have been born as a result, 14 of which were from using living donors. This demonstrates that uterine transplantation, using living donors, is viable option for women with uterine factor infertility.

The advantage of living uterus donation is that living donor organs are in better condition, and the uterus will only be without blood for a very short time after it is removed from the donor, which increases the chances of a successful transplant. Although there is no guarantee that any uterine transplant will work, the fact that 14 of the 15 successful livebirths following uterine transplant so far have been from living donors, exemplifies the potential advantages to using living donors. The success rates of other solid organs are also better from using living donors. Living uterus donation has the benefit of allowing the transplant operation to be scheduled at a time that is convenient for the recipient and the donor.

One of the most frequent concerns of potential living uterus donors is whether the loss of one uterus will impact on their health in later life. A healthy person can live a completely normal life without a uterus. Lifestyle is not affected, and normal work can continue. Therefore, it is possible to remove a uterus from a healthy living person and transplant it into someone who needs it, with no ill effects on the donor other than the operation itself.

Pre-operative assessment

There is a sequence of tests that are necessary to thoroughly examine the health of the potential donor as well as the wellbeing and anatomy of the uterus. These tests will be performed as an outpatient. Throughout the period of assessment, potential donors should bear in mind that these tests may reveal a reason for the uterus donation being unacceptable.

<u>Blood tests</u>
Blood samples will be taken for routine analyses. Haematology tests can show anaemia or signs of infection, and blood chemistry tests can determine uterus and liver function, or the suggestion of diabetes. Samples are also tested for hepatitis B and C, HIV, CMV, EBV and syphilis. Consent will be obtained before testing for HIV and counselling can be provided before and after the test. Another blood sample will be taken from both the donor and recipient to check that the recipient does not have 'antibodies' that may react against the donor. This is called cross matching.

<u>Urine analysis</u>
A routine urine test will be undertaken to assess for infection and any potential undiagnosed problems which may impact ability to donate.

Electrocardiogram (ECG)
This test is used to check that the heart is healthy and functioning properly. An ECG involves having several small electrosensitive pads placed at different points on your chest, arms and legs for a few minutes. You may wear your clothes or a hospital gown for the procedure. The pads monitor the electrical activity of your heart to produce a tracing. The pads will not cause any pain or give you an electric shock. If heart disease is present, an abnormal tracing may be seen, and this could increase the risks associated with anaesthesia during the hysterectomy.

X-rays
A chest X-ray may be taken to ensure there is no undiagnosed medical condition that may affect the lungs or heart.

Genito-urinary infection (GUM) screen / High vaginal swab
A routine genito-urinary screen including gonorrhoea, chlamydia and trichomonas vaginalis will be undertaken to ensure there is no infection. A swab will also be taken to look for other infections, or organisms, which may require treatment, such as thrush.

Cervical Smear
A cervical smear/HPV test is required to ensure there are no abnormal cells on the cervix, prior to transplantation.

Pelvic ultrasound
This is an ultrasound scan that checks the size and shape of the uterus, and can exclude any anatomical abnormalities. It is performed vaginally to ensure the best views are available.

CT and MR Angiography
A combination of both CT and MRI angiography will be used to reveal the number and size of blood vessels taking blood to and from the uterus, as well as further detail of the uterus itself. This is very important to ensure the surgeon can have detailed knowledge of the vessels that will be used to connect the uterus to the recipient. CT angiography is a special kind of X-ray taken of the abdomen. Iodine containing "dye" is then injected into a vein in the arm and the scan is repeated. A computer is then used to build a 3-dimensional view of the uterus as well as it's supplying and draining blood vessels. The whole procedure takes about 30-60 minutes. There is a risk of radiation exposure with the CT, which will be kept to a minimum, but may present a small increased risk of cancer in the future. MR angiography is a similar technique using a powerful magnet rather than X-rays.

The operation

The hysterectomy (removal of a uterus)

You will be admitted to hospital on the day of your operation. You will be seen by a nurse and be given stockings to wear to reduce the risk of blood clots in your legs. You will then be seen by the surgical team who will explain the operation in further detail and go through a consent form which you will complete once you are satisfied. A repeat blood test will be undertaken to ensure that, in the unlikely event of excessive bleeding, that we have matched blood available to give a blood transfusion if need be.

You will then be put to sleep under general anaesthesia. The surgeons will use a midline incision beneath your umbilicus (belly button), or a bikini line incision across the bottom of your abdomen. You will be given antibiotics as a precaution to reduce the chance of infection. You will also be given painkillers to ensure you do not wake up in pain. The operation includes the removal of the womb and surrounding tissues from the donor. This includes the cervix (neck of the womb), a small cuff of vagina, the

surrounding supporting ligaments, as well as the major blood vessels supplying and draining the uterus. The fallopian tubes will also be removed, as they are connected to the womb, but not implanted into the recipient, to reduce future risk of ectopic pregnancy. The ovaries will not routinely be removed but if you have gone through the menopause, this will be an option that is discussed with you depending on your wishes. The graft is lifted out of the wound, flushed with a cold solution to wash out blood and slow the metabolism, after which it is carried into the adjacent operating theatre where the recipient is waiting.

The incision is then sewn up in layers and she returns, via the recovery room, to the ward. Whilst the initial surgical technique resulted in an operation which took 12 hours, evolution of the surgical technique has resulted in an operation which takes 4-6 hours. You will have several temporary tubes or lines inserted during the operation. These may include a tube inserted into the bladder (called a catheter) and a drainage tube from the wound. Fluids can be administered through a drip and, because the incision can be painful afterwards, injections or infusions of painkilling drugs can be given, as required. Tubes are usually removed after the first one to three days when the donor is encouraged to get out of bed and sit in a chair. That way, the risk of complications can be minimised.

What are the risks of the operation?

The risks associated with this operation include anaesthetic risks, bleeding (including the possibility of receiving a blood transfusion), infection, venous thromboembolism (blood clot in veins in your legs or lungs) and damage to internal organs such as bowel, bladder, the tubes that connect the uterus to the bladder (ureters), blood vessels and nerves. There is also a small risk of the stitches coming away at the top of the vagina. There is a small risk of requiring a second operation in the event of complication.

What are the benefits to donating your womb?

The biggest advantage donors can enjoy is that they have given the gift of parenthood, which in turn creates new life. The feeling of satisfaction, which comes from donating a uterus to a someone, is immense and cannot be overestimated. Seeing someone enjoy a better quality of life because of your gift is very rewarding. This very positive aspect of living donation often outweighs the associated physical challenges.

What will happen after the operation?

You will wake up in recovery with a catheter in place and a drip in your arm. You will be transferred back to the ward where you will be monitored closely with frequent observations of your vital signs. You will be offered painkillers and kept comfortable whilst you recover. You will have a catheter in your bladder that will be removed the following day. You may also have a drain in place which reduces the risk of internal bruising. It is likely this will also be removed the day after the operation. You will also receive blood thinning injections and wear the compression stockings to help prevent a blood clot.

After your operation you will stay in hospital for 3-4 days. During this time, you will continue to receive pain killers, and symptomatic relief to facilitate your recovery. You will be helped to mobilise and discharged when you feel comfortable and safe.

How will you feel afterwards?

You will be asked to return to the hospital within the first few weeks after her operation to ensure that she has made a good recovery from the operation and that the wound has healed well.

Psychological effects

After donating a uterus, some people can feel quite emotional. There can be a sense of anticlimax; so much time has been spent thinking about the operation that life may seem a little empty afterwards. The donor may also feel sad and have an unconscious resentment towards the recipient if she feels unsupported by relatives and hospital staff after the operation, as attention is shifted to the recipient. This kind of feeling can be more pronounced if the recipient does not make good progress or the transplant is unsuccessful. In some cases, donors may need additional help and support, including counselling, which can be arranged. Counselling facilities are provided for the donor at some centres. The relationship between donor and recipient and the impact of donation will be individual to each pair. People who receive a uterus are always grateful, but they are unable to repay the gift. So, it is important to avoid reminding them of their 'debt'. The donor may be able to help by maintaining a normal relaxed attitude towards the recipient.

Getting back to a routine

The first three months after a transplant is the 'settling down' period and when most problems tend to occur. Once these three months have passed, both the donor and the recipient can start to resume a normal routine. Depending on their work or lifestyle commitments and the type of surgery, donors can expect to be at home recuperating after the operation for up to 8 weeks. Sometimes this can be a frustrating time, wanting to return to a normal life, but without the energy and overall health. Patience is required, as is support from other family members. The donor should allow between 6-8 weeks to get back to full activity If the donor regularly sees the recipient, this can be an added source of satisfaction – watching the recipient return to good health can ease some of the possible negative feelings.

Driving

The Driver and Vehicle Licensing Agency (DVLA) has no hard and fast rules with regard to starting to drive again. Generally, if the donor feels well and capable, she can usually return to driving after four to six weeks. Car insurance should be checked, as the length of time after an operation that you are not insured to drive varies depending on your policy.

Exercise

Maintaining a healthy lifestyle is as important after donation as beforehand. A post-donation exercise programme should begin slowly, with the length of time spent exercising and the effort involved being increased over a period of time.

Sexual relationships

Donors should be able to resume their usual sexual relationships as soon as they feel comfortable. To allow healing of the vaginal vault, a period of 4-6 weeks abstinence should be adopted. It may take a few months before normal activities can be undertaken, but this depends on the particular individual's recuperation.

Appendix S2. Questionnaire (V1 - 21/04/2018)

Full title: Motivations and attitudes amongst women offering to donate their womb for womb transplantation

Please complete the following questionnaire after reading the participant information sheet (V2 30-4-19).

Consent

1. I confirm that I have read and understand the participant information leaflet (V1 21-04-19) and have had the opportunity to consider the information, ask questions and had these answered satisfactorily. ☐

2. I understand that my participation is voluntary and that I am free to withdraw at any time without giving any reason, without my legal rights being affected. ☐

3. I understand that the information collected may be used to support other research in the future, and may be shared anonymously with other researchers. ☐

4. I agree to take part in the above study. ☐

1. Demographic information

a) Age (years)

16-19

20-29

30-39

40-49

50-59

60+

Would rather not say

b) Ethnicity

White

Asian

Black

Mixed

Other

Would rather not say

c) **Employment status**

Employed (Full time)

Employed (Part time)

Self Employed

Student

Housewife

Unemployed

Would rather not say

a) **Religion**

Christian

Muslim

Hindu

Other

Athiest

Would rather not say

b) **Relationship status**

Single

Living with partner

Married

Divorced

Separated

Widowed

Would rather not say

2. **Medical history**

Height (cm):

Weight (kg):

Do you have any medical problems we should be aware of? Yes ☐ No ☐

If yes, please detail below:

Have you previously had cancer? Yes ☐ No ☐

If yes, please detail below:

Do you have HIV, hepatitis or syphilis? Yes ☐ No ☐

If yes, please detail below:

Have you been diagnosed with endometriosis or adenomyosis? Yes ☐ No ☐

If yes, please detail below:

Do you have any psychiatric problems we should be aware of? Yes ☐ No ☐

If yes, please detail below:

3. Surgical History

Have you had any previous operations inside your abdomen, in particular on your womb or cervix? Yes ☐ No ☐

If yes, please provide details:

Year	Operation	Complications	Additional comments

4. Childbearing history

Have you had children before?

Yes No

If yes: How many children have you had?

0 1 2 3 4+

Were any of your babies delivered by Caesarean section?

Yes No

If yes: How many?

0 1 2 3 4+

Did you have any of these problems in any of the pregnancies?

Pre-term delivery (<37 weeks) | Pre-eclampsia
Heavy bleeding after delivery | Gestational diabetes
Obstetric cholestasis | Not applicable

If so, please explain further…

Have you completed your family?

Yes No

5. Gynaecological history

Have you had any previous miscarriages?

Yes No

If yes: how many miscarriages have you had?

1 2 3+

Have you previously had an abnormal smear?

Yes No

If yes: did you have any surgical treatment to remove the abnormal cells?

Yes No

If yes: have you had a normal smear since?

Yes No

Are you still having regular periods?

Yes No

6. Social history

Occupation:

What is your main language?

If your main language is not English, are you fluent in English?

Yes ☐ No ☐

Do you smoke? Yes ☐ No ☐

If yes, how many cigarettes do you smoke a day?

Have you smoked previously? Yes ☐ No ☐

If yes,

How many years did you smoke for?

On average how many cigarettes did you smoke per day?

When did you give up smoking?

Do you drink alcohol? Yes ☐ No ☐

If yes, how many units of alcohol so you consume a week?

7. **Knowledge about womb transplantation**

I believe that adoption and surrogacy are suitable methods to have children for women who do not have a functional womb

Strongly agree Agree Undecided Disagree Strongly Disagree

If disagree/strongly disagree, why? ………..…………………………………………

How much do you know about womb transplantation?

A lot A fair amount Heard it discussed only a few times Nothing

I understand the benefits of womb transplantation.

Strongly agree Agree Undecided Disagree Strongly Disagree

If disagree/strongly disagree, why? ………..…………………………………………

I understand the risks of donating my womb

Strongly agree Agree Undecided Disagree Strongly Disagree

If disagree/strongly disagree, why? ………..…………………………………………

I am aware that if I donate my womb, I will be unable to have further children.

Yes No

I am aware that if I donate my womb, my ovaries will not be removed, so I will continue to have normal hormones

Yes No

I understand that if I donate my womb, my ovaries will not be removed, so any future babies born in the womb after donation will not be genetically related to me.

Yes No

I understand that after I donate my womb, I would no longer have periods

Strongly agree Agree Undecided Disagree Strongly Disagree

If disagree/strongly disagree, why? ……….…………………………………………

I believe that not having periods would improve my quality of life

Strongly agree Agree Undecided Disagree Strongly Disagree

If disagree/strongly disagree, why? ……….…………………………………………

8. Motivations for womb donation

Please indicate if you agree/disagree with the following statements:

I want to donate my womb to help someone else carry and give birth to their own baby

Strongly agree Agree Undecided Disagree Strongly Disagree

I know someone personally who is infertile who I would like to donate my womb to

Strongly agree Agree Undecided Disagree Strongly Disagree

I want to donate my womb to help others

Strongly agree Agree Undecided Disagree Strongly Disagree

I want to donate my womb to help contribute to science

Strongly agree Agree Undecided Disagree Strongly Disagree

I want to donate my womb because I am already an organ donor and want to donate another organ

Strongly agree Agree Undecided Disagree Strongly Disagree

I want to donate my womb because I previously was or considered being a surrogate

Strongly agree Agree Undecided Disagree Strongly Disagree

I want to donate my womb because I no longer need it

Strongly agree Agree Undecided Disagree Strongly Disagree

I believe that the potential benefits of donating my womb to help an infertile woman give birth outweigh the potential risks of the surgery

Strongly agree Agree Undecided Disagree Strongly Disagree

If disagree/strongly disagree, why? ……….…………………………………………

I believe that women should be freely able to voluntarily donate their womb for womb transplant, as long as they are fully informed

Strongly agree Agree Undecided Disagree Strongly Disagree

If disagree/strongly disagree, why? ……….…………………………………………

I would be happy to undergo the necessary pre-operative investigations to ensure my womb is safe for transplantation

Strongly agree Agree Undecided Disagree Strongly Disagree

If disagree/strongly disagree, why? ……….…………………………………………

If you are menopausal, would you be accepting of taking hormone replacement therapy for 3-6 months pre-operatively. This will give you monthly withdrawal bleeds to help demonstrate that the lining of your womb is still functional.

Strongly agree Agree Undecided Disagree Strongly Disagree

Not applicable

If disagree/strongly disagree, why? ……….…………………………………………

I understand and accept the expected recovery period of 3-4 days in hospital and up to 8 weeks before being fully back to normal

Strongly agree Agree Undecided Disagree Strongly Disagree

If disagree/strongly disagree, why? ……….…………………………………………

I understand that I would not receive any payment for donating my womb

Strongly agree Agree Undecided Disagree Strongly Disagree

If disagree/strongly disagree, why? ……….…………………………………………

If you donated your womb for transplantation, would you want to be informed of subsequent progress of recipient, for example to find out that they have become pregnant or had a baby?

Strongly agree Agree Undecided Disagree Strongly Disagree

If disagree/strongly disagree, why? ……….…………………………………………

Are you aware that the woman who receives a womb transplant is at greater risk of cancer and infection because of the need to take immunosuppressive medications after the transplant?

Strongly agree Agree Undecided Disagree Strongly Disagree

If disagree/strongly disagree, why? ……….…………………………………………

Does knowing about the additional risks of cancer and infection on the woman who receives a womb impact your perceptions on womb transplantation?

Strongly agree Agree Undecided Disagree Strongly Disagree

If strongly agree or disagree, why? ………..…………………………………………

Given the novelty of the operation, and the media interest in the process, I understand there is a small risk of unwanted media intrusion if I went ahead with donating my womb

Strongly agree Agree Undecided Disagree Strongly Disagree

If disagree/strongly disagree, why? ………..…………………………………………

Having read the patient information sheet, and considered the risks, I am still keen to donate my womb for transplantation.

Strongly agree Agree Undecided Disagree Strongly Disagree

If disagree/strongly disagree, why? ………..…………………………………………

Any other comments to add?

Thank you for completing this questionnaire and taking part in this study
